# Mastoid Cavity Obliteration with Combined Palva Flapand Bone Pâté

**Published:** 2015-01

**Authors:** Samad Ghiasi

**Keywords:** Bone pâté, Chronic otitis media, Cholesteatoma, Mastoidectomy, Mastoid obliteration, Palva flap.

## Abstract

**Introduction:**

This study was designed to evaluate the usefulness of mastoid cavity obliteration with combined bone pâté and Palva flap in the prevention of problematic mastoid cavities after canal wall down mastoidectomy.

**Materials and Methods:**

In a prospective longitudinal study with a mean follow-up of 28 months conducted between 2008–2012, a series of 56 ears in 48 patients with chronic otitis media due to a cholesteatoma underwent canal wall down mastoidectomy that their mastoid cavity obliterated with combined bone pâté and Palva flap. Seventeen (30%) ears were managed via revision surgery, with the reminder via primary surgery. Data included mastoid cavity status, results at second-look surgery with ossiculoplasty, and postoperative complications.

**Results:**

All patients underwent second-look surgery. Forty-six (82%) ears maintained a very small, dry and healthy mastoid cavity. Seven (13%) ears had occasional otorrhea, and three (5%) ears had small granulation tissue. Seven (12.5%) ears had residual cholesteatoma pearl in the middle ear at second-look surgery. Four (7%) ears exhibited wound infection.

**Conclusion:**

Canal wall down mastoidectomy and mastoid cavity obliteration with combined bone pâté and Palva flap is a effective option for the complete removal of cholesteatoma and prevention of postoperative mastoid cavity problems.

## Introduction

Chronic otitis media (COM) with cholesteatoma is a common disease in otology. The principal goal in cholesteatoma surgery is the complete eradication of the disease to produce a dry, safe and self-cleaning ear and creation of new anatomy to prevent recurrence. Canal wall up (CWU) techniques have many advantages such as preserving the posterior canal wall, eliminating the need for periodic bowl cleaning, avoiding the risk of recurrent bowl infections, and simplifying ossicular reconstruction. However, in CWU techniques, the relapse rate of cholesteatoma is as high as 36% in adults and 67% in children (1).

Canal wall down (CWD) mastoidectomy is a well-established surgical technique used to completely remove cholesteatoma. The advantages of CWD mastoidectomy include excellent exposure of the entire attic and middle ear and complete eradication of disease. Using this technique the relapse rate could be as low as 2% (1,2). However, this technique has some disadvantages, such as accumulation of debris in the exteriorized mastoid cavity, a requirement for periodic cleaning and water restrictions to prevent bowl infection, and problems relating to ossicular reconstruction, non-aesthetic meatoplasty, reports of vertigo in cold weather and during swimming, and difficulty with fitting a hearing aid (1,2).

Recently mastoid obliteration has been used in CWU tympanoplasty for cholesteatoma to facilitate tympanic aeration and ultimately to prevent future recurrence of cholesteatoma (3). A CWU or CWD technique is not suitable for use in all ears with COM, but most surgeons choose one of the techniques based on the middle ear cleft pathology. The principal advantages of mastoid cavity obliteration are 1) reduced nitrogen-absorbing mucosa in the mastoid cavity preventing recurrence of retraction cholesteatoma in patients with eustachian tube dysfunction, 2) elimination of mastoid cavity preventing accumulation of squamous epithelium and bowel infection (1-3).

The size of the surgical cavity can be diminished using obliteration to create a small cavity that is self-cleaning and easily maintained. Both autologous and synthetic materials have been used for obliteration. Materials such as free graft, fat, cartilage, bone chips, bone pâté, hydroxyapatite, and periostio-muscular flaps are used (4-7). In this study we obliterated the mastoid cavity with bone pate and a Palva flap. This communication describes the surgical technique, results, and management of complications.

## Materials and Methods

A prospective longitudinal study of patients who underwent CWD mastoidectomy between 2008–2012 at the Tabriz University of Medical Sciences, Iran was performed. All procedures were performed by the author. A database was designed to record pertinent data including age, sex, postoperative status of the mastoid cavity, tympanic membrane and findings at second-look surgery including presence of residual disease. Occurrence of operative cavity pathology and recurrence of cholesteatoma were recorded.

***Surgical Technique***

**Fig 1:**
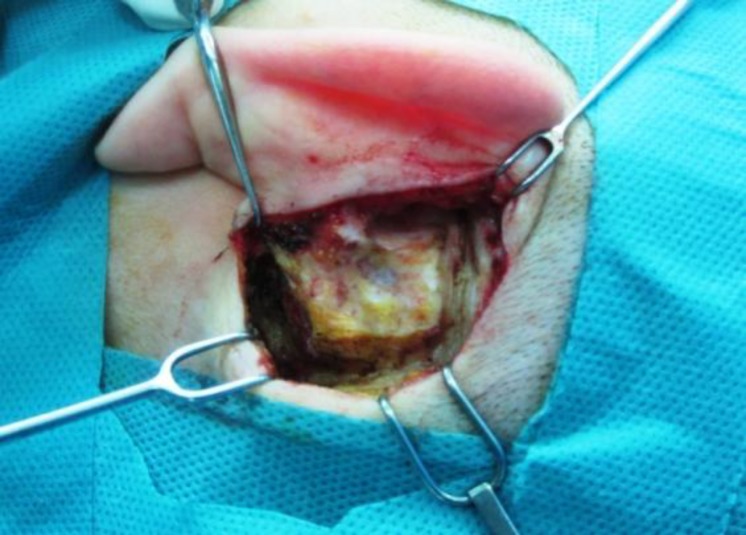
Palva flap

The procedure was performed under general anesthetic using a postauricular approach. A Korner skin flap and a meatally-based, wide musculoperiosteal flap (Palva flap) (approximately 4×4 cm) was prepared (Fig. 1) (8).

**Fig 2:**
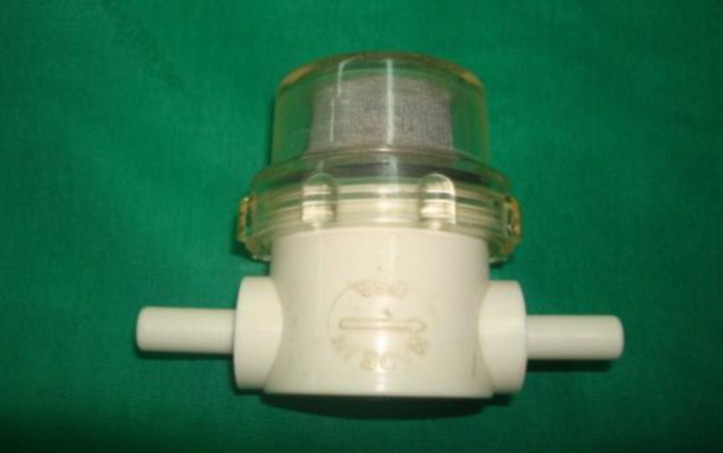
Bone pâté collector

**Fig 3:**
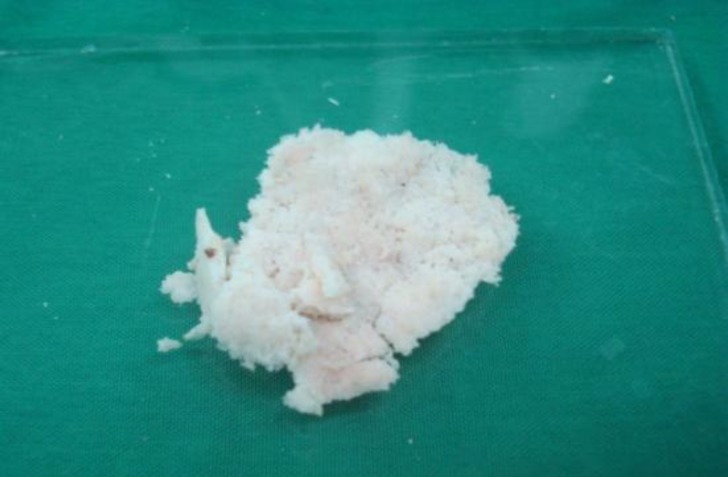
Collected bone pâté

A Sheehy bone pate collector (Otomed, Lake Hvasu city, AZ) was used to collect bone pâté from the mastoid and squamosa cortex of the temporal bone (Fig.2), stopping before exposure of the mastoid air cells. Bone pate was then mixed with antibiotic and steroid and set aside (Fig. 3).

**Fig 4:**
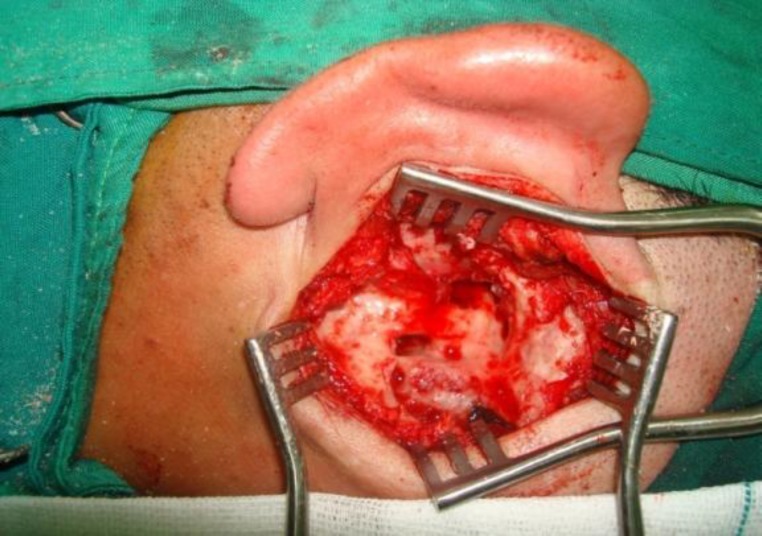
Canal wall down mastoidectomy

A complete mastoidectomy with removal of posterior wall was performed (Fig.4). 

The cholesteatoma sac, pathologic mucosa, incus, malleus head and tensor fold were removed. A flask-shaped tear drop of silastic sheeting (0.04-inch thickness) was placed in the tympanum with the taper of the silastic in the eustachian tube entrance and the remainder maintaining the middle space. A large fresh temporalis fascia graft was used in an underlay fashion to reconstruct the tympanic membrane. A small meatoplasty was then performed to promote healing and self-cleaning of the new reconstructed wide external canal. 

**Fig 5:**
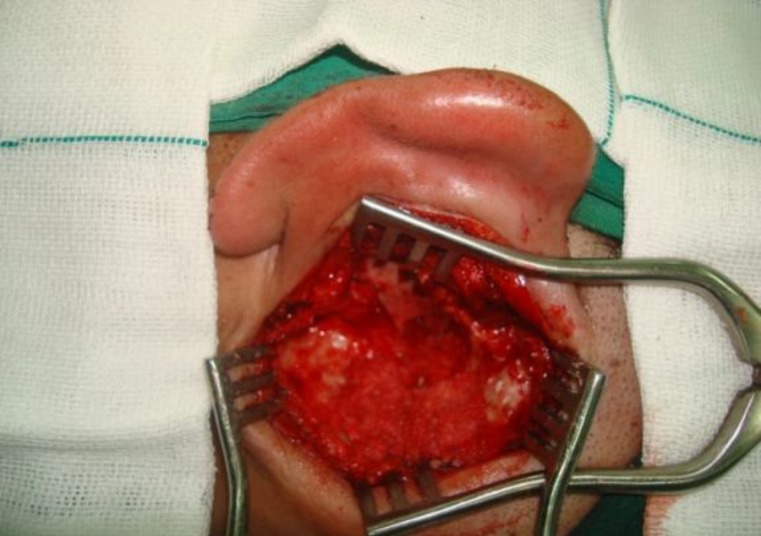
Obliterated mastoid cavity with bone pâté

 The mastoid, antrum and epitympanic space posterior to the cochleariform process and superior to the tympanic segment of the facial nerve and up to the level of the mastoid cortex was obliterated with bone pâté (Fig.5) and covered by the posterior section of the temporalis fascia graft. 

The Palva flap was placed into the mastoid cavity, over the temporalis facia graft, and the Korner flap was placed on top. The external auditory canal was packed with Gelfoam and rosebud. A quarter-inch Penrose drain was placed lateral to the obliterated mastoid cavity and the wound was closed using Vicryl 3-0 subcutaneously in one layer, and a standard mastoid dressing was applied.

Intravenous antibiotic (Cefteriaxone) was administered continuously for 48 hours and intramuscularly for another 5 days. Patients used oral Cefixime to complete the 14-day course. The Penrose was removed on Day 2 following surgery. The mastoid dressing was changed daily until discharge, and the rosebud was removed after 14 days.

Second-look surgery with ossiculoplasty was performed, typically after 6 months among adults and 12 months in children after the initial tympanomastoidectomy. During second-look surgery, the status of the mastoid cavity, and tympanic membrane graft were assessed. The middle ear was investigated for the presence of residual cholesteatoma, and ossicular reconstruction was performed.

## Results

Fifty-six ears in 48 adult and children were subjected to CWD mastoidectomy with mastoid cavity obliteration. Table. 1 presents the patient demographics.

**Table 1:** Patient Demographics

**Case**	
Total patients	48
Total Ears	56
Mean Age(years)	28(8-56)
Male	21(44%)
Female	27(56%)
<18 years	4(8%)
Previous Ear Surgery	17(30%)

Eight patients had bilateral surgeries performed on different occasions. Seventeen (30%) ears were managed via revision surgery, with the remainder via primary surgery. The mean postoperative follow-up time was 28(8-42) months. Forty-six (82%) ears maintain a very small, dry and healthy mastoid cavity. Seven (13%) ears had occasional otorrhea that was relatively easily managed by topical therapy, and three (5%) ears had small granulation tissue that was treated by silver nitrate. Four (7%) ears exhibited wound infection and were treated intravenously with antibiotics. In all patients there was ossicular destruction that required reconstruction. Second-stage surgery was performed in all patients for detection of cholesteatoma recurrence and for ossicular reconstruction. Seven (12.5%) ears had evidence of residual keratin pearl in the middle ear at second-look surgery that was easily resolved.

## Discussion

Unlike CWU techniques, traditional CWD techniques involving removal of the posterior canal wall improves exposure and facilitates the complete removal of all cholesteatoma. However, despite the fact that most well-constructed mastoid cavities remain problem-free, they do require periodic cleaning and are prone to bowl infection. Preservation of the posterior canal wall results in a higher rate of recidivism, particularity in children, while CWD techniques also provide for removal nitrogen-absorbing mucosa of the mastoid. After surgery, the new epithelial lining of the mastoid bowl is stratified keratinizing epithelium (1). CWU techniques are associated with an intermediate risk for otorrhea but an increased risk of cholestea- toma recurrence (30–63%). In contrast, the risks are reversed with CWD techniques, with a lower risk of recurrence (2–10%) but higher risk of otorrhea (20–60%) (7). 

Mastoid cavity obliteration could reduce problems of a large mastoid cavity. A range of materials has been used by many otologists to obliterate the mastoid cavity using various procedures including free grafts-biologic techniques (bone pâté, allogenous/autogenous bone chip, cartilage, fat, and fascia); free grafts – non-biologic techniques (hydroxyapatite, calcium phosphate ceramic granules), bioactive glass ceramic, and silicone bocks); local flaps (Palva, Hong Kong, middle temporal artery, temporoparietal fascia, pedicled superficial temporal fascia, postauricular-periosteal-pericranial, temporalis muscle, inferiorly based fascioperiosteal (19), postauricular myocutaneous, and composite multi-fracture osteoperiosteal (9-21)).

The greatest advantage of this technique is its technical simplicity. In this procedure, a combination of biologic graft and local flap is used. In this technique, first the mastoid cavity is obliterated with bone pâté and is shaped smoothly, then a Palva flap is placed into the mastoid cavity over the new reconstructed cavity. The Palva flap, in turn, provides a suitable vascular bed to allow regrowth of skin from the Korner flap to the new reconstructed mastoid cavity. The early resolution of the wound is another advantage of this combined procedure. A Palva flap in the mastoid cavity prevents exposure of the obliterated material and necrosis of posterior canal wall (Korner flap) skin. Takahashi et al reported exposure of the obliterated material due to necrosis of the soft posterior wall skin when they used only bone pâté to obliterate the mastoid cavity (10). Hormann et al reported lower a reduced risk of epithelization and Takahashi et al documented a higher percentage of exposure of obliterated material when an apatite ceramic was used (22,23). 

When muscular flaps are used alone to obliterate the mastoid cavity, the muscle becomes atrophied and the mastoid cavity becomes larger. A combined bone pâté and Palva flap procedure prevents the early complication of posterior canal wall skin necrosis and exposure of the obliterated material and enlargement of the mastoid cavity. The only limitation associated with mastoid obliteration is the requirement for the surgeon to ensure complete removal of the cholesteatoma matrix from the mastoid cavity.

In our study, this technique had acceptable results, with a high percentage (82%) of ears maintaining a dry, safe and self-cleaning small mastoid cavity. A few problems remained in 18% of cases which were resolved in the outpatient service. The only complication encountered using this technique was postoperative wound infection when intravenous antibiotics were used for 2 days. All infections occurred among the first patients to undergo the procedure. Therefore we modified our protocol to include a total of 2 days’ perioperative intravenous followed by 5 days of intramuscular antibiotics. No cases of postoperative infection were observed under the modified protocol. This procedure has been successful in cases of cholesteatoma complicated with lateral semicircular canal fistula. Using a small meatoplasty, the newly formed mastoid cavity has provided sufficient draining and aeration, an improved aesthetic appearance, and the provision for a hearing aid to be fitted comfortably. However, long-term follow-up (ideally for 10 years) will be needed in order to investigate the occurrence of residual cholesteatoma under the obliteration material or tympanic membrane retraction. 

## Conclusions

In modern ear surgery, mastoid cavities due to CWD mastoidectomy are obliterated using various techniques and materials. CWD mastoidectomy and combined bone pâté and Palva flap mastoid cavity obliteration is a technique that facilitates exposure of the middle cleft and ensures complete removal of cholesteatoma. In our experience, this procedure is an effective method to manage patients with pre-existing mastoid cavities and also those not previously operated upon. The outcome in all cases was a safe, dry, and self-cleaning ear. 
